# Seasonality and Sleep: A Clinical Study on Euthymic Mood Disorder Patients

**DOI:** 10.1155/2012/978962

**Published:** 2011-12-08

**Authors:** Chiara Brambilla, Chiara Gavinelli, Dario Delmonte, Mara Cigala Fulgosi, Barbara Barbini, Cristina Colombo, Enrico Smeraldi

**Affiliations:** Department of Neuropsychiatric Sciences, Scientific Institute, University Vita-Salute San Raffaele, 20127 Milan, Italy

## Abstract

*Background*. Research on mood disorders has progressively focused on the study of seasons and on the mood in association with them during depressive or manic episodes yet few studies have focused on the seasonal fluctuation that characterizes the patient's clinical course both during an illness episode and during euthymic periods. *Methods*. 113 euthymic outpatients 46 affected by major recurrent depression and 67 affected by bipolar disorder were recruited. We evaluated the impact of clinical “rhythmical” factors: seasonality, sleep disturbance, and chronotype. Patients completed the SPAQ+ questionnaire, the MEQ questionnaire, and the medical outcomes study (MOS) sleep scale. We used *t*-test analyses to compare differences of clinical “rhythmical” and sociodemographic variables and of differences in the assessment scales among the diagnostic groups. *Results*. Patients reporting a family history for mood disorders have higher fluctuations throughout seasons. Sleep disturbance is more problematic in unipolars when compared to bipolars. *Conclusions*. Sleep, light, and seasonality seem to be three interconnected features that lie at the basis of chronobiology that, when altered, have an important effect both on the psychopathology and on the treatment of mood disorders.

## 1. Introduction

The degree to which seasonal changes affect mood is known as seasonality. The periodical pattern of recurrence is a biological feature of mood disorders, and the recovery from the first episode of illness is followed by a subsequent recurrence in about 90% of affected patients during their lifetime [1]. In addition, the occurrence of diurnal variations in mood and of distressful and pervasive disruption in sleep suggests a primary disturbance of biological rhythms for mood disorders [2]. 

The typical pattern of recurrence of mood disorder follows interindividual rules, since each patient presents their own specific pattern with a few episodes during their life, or more than four episodes each year (rapid cycler). Some authors have stressed the concept of seasonality, as many patients show critical months of the year when they tend to have a new recurrence independent of polarity [3]. The recurrence pattern is also affected by external factors, such as exposition to light which appears to have an antidepressant effect both on its own and when associated to acute pharmacological treatments, which shorten the subsequent cycle of illness [4] and maintenance treatments, which are expected to decrease the episode rates. Mood stabilizers, the first choice of treatment for bipolar disorder, can successfully sustain the effect of chronotherapeutics and can help to manage the low risk of manic switches [5]. Chronotherapeutic treatments such as sleep deprivation have been successfully associated with antidepressant drugs with a serotonergic [6–8] noradrenergic [9], mixed serotonergic-noradrenergic [10–12], and dopaminergic [13] mechanism of action. In a similar way, light therapy has been shown to hasten response to serotonergic antidepressants in nonseasonal depression [8, 14]. Therefore, an environmental regulator of fundamental importance on biorhythms in living organisms is represented by the photoperiod, which is the variability of hours of light throughout seasons. The disruption of circadian rhythms, including an abnormal response to sunlight, have a role in the pathophysiology of mood disorders; consequently, both climate and latitude may influence seasonality [15]. On the extreme end of the spectrum, such as with seasonal affective disorder (SAD), the change of symptoms with the seasons is associated with significant dysfunction. Yet, even patients with mood disorders not formally diagnosed as SAD can show seasonal worsening of their symptoms [16] meaning that seasonality can be considered as a continuum.

What is the nature of sleep disorders in major depression? It has been hypothesized that the circadian type, in other words, a person's chronotype, may have a role in seasonal susceptibility in morningness and eveningness types [17]; morningness types maintain a more regular sleep-wake cycle and prefer to carry out its activities during the day time, while eveningness types have a more flexible sleep-wake cycle and are more active in the evening hours. Natale et al. (2005) found that evening types seem to feel better in summer (long photoperiod) than in other seasons, while morning types seem to feel better in winter (short photoperiod) [18]. It can be hypothesized therefore that the duration of the photoperiod and its variations throughout the seasons may influence the relapse or the reoccurrence of an illness episode on the basis of the subject's chronotype. Furthermore, Perlman and colleagues (2006) found that a persistent sleep deficit after recovery (at least partially) from a mood episode in Bipolar patients predicted depressive symptoms during a six month follow-up period [19]. 

### 1.1. Genetic Factors

Various twin studies suggest that there is a genetic component behind susceptibility to seasonal changes. A large study involving 4,638 adult twin pairs from Australia found that genetic factors accounted for 29% of the variance in seasonality. They also found that in each behavioral domain, (i.e., mood, energy, social activity, sleep, appetite, and weight), there is a significant genetic influence on the reporting of seasonal changes [20]. Another twin study involving 339 twin pairs [21] found that genetic factors accounted 45.5% in males and 30.5% in females of the variance for seasonality. 

## 2. AIM

The purpose of this clinical study was to evaluate the effect of clinical “rhythmical” factors such as seasonality, sleep disturbance, and chronotype, which can have an influence on the course of the patient's illness during a period of euthymia. We obtained information on their seasonal and sleep disturbances during a period of euthymia and compared these variables between unipolars versus bipolars. We compared patients with or without family history of mood disorders for the variable “seasonality”, we compared morningness and eveningness chronotypes to assess differences in sleep quality and at last, we evaluated the effect of a lithium maintenance therapy on chronobiological parameters comparing the patients with or without lithium salts.

## 3. Methods

### 3.1. Participants

The sample in this cross-sectional longitudinal study included 113 outpatients (80 females and 33 males): 46 affected by major depressive disorder (unipolars) and 67 affected by bipolar disorder. The sample was recruited during the patients checkups at the outpatient Mood Disorder Unit of the San Raffaele Hospital in Milan, from January, 2009 to March, 2010. Inclusion criteria were (1) bipolar or unipolar disorder diagnosis according to DSM-IV-TR criteria, (2) euthymic patients which had a normal ranged mood, without a depressed or elated mood for a period of at least 24 months as measured by the hamilton depression rating scale (HDRS) <8 or the young mania rating scale score = 0, and (3) lithium or antidepressant (SSRIs) maintenance therapy, according to the polarity of the forms and clinical judgment. The lithium dosage was given on the basis of the tested plasma level between 0.5 and 0.7 mmol/L.  Table 1 summarizes the clinical and demographic characteristics of the sample. This study has been approved by the local sanctioning board (ASL, Città di Milano), and it meets ethical standards. 

### 3.2. Measures

The protocol for data collection consisted of an anamnestic sheet filled out by the psychiatrist during the patient's checkup, and in a series of self-administered questionnaires: *seasonal pattern assessment questionnaire (SPAQ), morningness eveningness questionnaire (MEQ),* and *medical outcomes study (MOS) sleep scale*.

In the anamnestic sheet, the following data was collected: age, sex, the polarity of the mood disorder, other Axis II and III diagnoses (according to the DSM IV-TR), the age of onset, total of number episodes, the recurrence index (number of previous episodes/duration of illness), years of education, family history of mood disorders, the current therapy, and the duration of euthymia duration of maintenance therapy. Most bipolar patients were on a monotherapy long-term treatment with lithium salts. 

#### 3.2.1. Seasonal Pattern Assessment Questionnaire (SPAQ+)

The SPAQ+ (Italian version, [22]) is a self-administered questionnaire which quantifies (regardless of the presence or absence of a psychiatric disorder), the individual's tendency to seasonal mood and behavioral changes, and this is defined as “seasonality.” The SPAQ [23] investigates several areas including demographic data, seasonal changes in sleep length, social activity, mood, weight, appetite, and energy level (Likert scales scored 0–4, used to calculate the global seasonality score or GSS, with a total score ranging from 0–24).

#### 3.2.2. Morningness-Eveningness Questionnaire (MEQ)

Morningness types are those people who consistently prefer diurnal activity, while eveningness types are those who prefer nocturnal activities. The morningness/eveningness dimension is regulated by a complex interaction among social, geographic, and genetic factors. The morningness/eveningness questionnaire [24] is the most commonly used self-report questionnaire for the assessment of preferred timing of complex behaviors regarding sleep-wake habits and rhythms. The questionnaire is normally distributed [25], and this allows us to consider the circadian types as a continuum [26]. The Italian version of the MEQ [27] includes 19 items, and the sum yields a global score, ranging from 16 to 86 with lower scores indicating greater evening tendencies and higher scores indicating greater morningness tendencies.

#### 3.2.3. Medical Outcomes Study (MOS) Sleep Scale

The MOS [28] is a self-administered instrument which consists of 12 items which measure six important dimensions of sleep, including initiation (time to fall asleep), sleep disturbance (have trouble falling asleep, how long to fall asleep, sleep was not quiet, awakenings during sleep and having trouble falling back to sleep), quantity (hours of sleep each night), maintenance, respiratory problems, perceived sleep adequacy (get enough sleep to feel rested upon in the morning, getting the needed amount of sleep), and day-time somnolence (drowsiness during the day, nap taking). Answers were based on a retrospective assessment over the past 4 weeks. 

### 3.3. Statistical Analysis

A descriptive statistics of the sample was carried out using SOFTSTA 6.0. In accordance with diagnosis, gender, chronotype, family history, maintenance therapy non paired Student's *t*-tests were carried out for the sociodemographic, clinical variables, and rating scale scores (Table 1). 

## 4. Results

We then performed a *t*-test analysis to compare the two different disorders, unipolar and bipolar patients, and found a significant difference for greater sleep disturbance in unipolar patients (*t* = −2.27; *P* = 0.003). There is a significant difference also in the recurrence index, with a higher recurrence in bipolar patients (*t* = −0.55; *P* > 0.000) (Table 2). 

We performed a *t*-test analysis between patients that reported a family history for mood disorders, and we found significant difference in the following variables: age of onset (*t* = 3.74; *P* < 0.000) which is lower in individuals with a family history, duration of illness (*t* = −3.25; *P* < 0.001) which is longer for patients with a family history, number of depressive episodes (*t* = −2.66; *P* = 0.009) and total number of episodes (*t* = −2.58; *P* = 0.011), which are higher for patients with a positive family history and GSS (*t* = −2.13; *P* = 0.035) which is higher for patients with a positive family history (Table 3). 

We decided to analyze if lithium salts (the maintenance therapy considered a gold standard in the treatment of bipolar disorders) when compared to other therapies, has an effect on the variables analyzed earlier as its effects on sleep quality have not previously been taken into consideration in literature. In our sample of unipolar and bipolar patients, we performed a *t*-test analysis between patients that take lithium in their maintenance therapy for at least 6 months versus patients that do not. There were significant differences for sleep disturbance (*t* = 3.03; *P* = 0.003), shortness of breath (*t* = 3.83; *P* < 0.000), sleep adequacy (*t* = 3.63; *P* < 0.000), and a notable even if not significant difference in sleep somnolence (*t* = 1.79; *P* = 0.060) indicating that these sleep quality parameters better in those patients that use lithium salts as a maintenance therapy (Table 4). 

We also divided subjects according to their chronotype to verify if being a morningness versus eveningness type would influence our variables. The chronotype division was performed using the subjects' MEQ scores. We performed a *t*-test analysis dividing MEQ according to categories of chronotype into greater “morningness” (>59) and greater “eveningness” (<41) and intermediate (>41 and <59) [26]. Patients highlighted a significant difference in sleep disturbance (*t* = 2.52; *P* = 0.010) with morningness types reporting a lower sleep disturbance and a better sleep adequacy (*t* = 2.53; *P* = 0.013) (Table 5). 

## 5. Discussion 

 Seasonality seems to have a tie with a family history for mood disorders: patients (both unipolar and bipolar) reporting a positive family history for their disorder have a significantly higher seasonality, indicating that there might be a genetic component that influences the amount of mood, energy, appetite and sleep fluctuation throughout seasons. This data is consistent with the findings of Jang et al. [21] who found that a genetic factors accounted for 45.5% (males) and 30.5% (females) of the variance. Future genetic studies on the heritability of seasonality could support further confirmations of these findings. 

Patients that take lithium salts as a maintenance therapy have a lower sleep disturbance, a lower shortness of breath, a better sleep adequacy and an overall improved sleep quality. This may suggest that lithium salts aid in stabilizing not only mood fluctuations themselves, but also help to regulate all the rhythmical parameters such as sleep rhythms, which are directly involved with the pathophysiology of mood disorders. This could be a possible explanation to the fact that unipolar patients reported more sleep problems, as the majority of them do not take lithium Salts (38 do not take it out of 46) as opposed to most bipolar patients who do (50 take it out of 57) In a previous study, our group [13], discussed the benefits of lithium salts on sleep. A possible explanation is that lithium is known to delay circadian rhythms in animals [29] and lengthen the period of the sleep-wake cycle in humans [30], leading to a phase delay of circadian rhythms both in normal subjects and in bipolar patients [31]. Given the effects of lithium on the rhythmicity of brain neurotransmitter function [32] and recent findings about the regulation of circadian rhythmicity in mammals [33, 34], the chronobiological effect of lithium has been proposed to at least partially explain its clinical effect [35, 36].

When we subdivided our patients according to chronotype, we found morningness patients to have a sleep rhythm which is less disturbed and a more adequate sleep quality. Hätönen et al. [37] and Mansour et al. [38] have found that circadian-type preference for evening activities has been shown in bipolar patients: eveningness seems to be related to morbidity and especially to sleep and mood disorders [39]. It has been hypothesized that mood disorders may be more prevalent in individuals with a shifted clock. One hypothesis is that clock genes have been shown to regulate circadian behaviour and preference [40], so sleep and rhythm performance may be a part of the phenotype corresponding to these circadian clock shifts [41]. Another possible explanation is that since exposure to outdoor light affects the phase of entrainment and our society has schedules for the most part synchronised according to morningness types, eveningness types pay the consequences in terms of sleep loss and reduced sleep quality [42]. It has been shown that repeated sleep restriction causes a cumulative increase in daytime sleepiness and waking neurobehavioral deficits [43]. This is a relevant issue especially for mood disordered patients, because altered sleep quality can be one of the triggers for an illness episode. As it is widely known, sleep loss is often a precursor and/or a precipitant of hypomania or mania in bipolar patients. Knowing a patient's chronotype can be useful in helping the patient to maintain a regular sleep hygiene to prevent sleep loss and consequently a circadian rhythm deregulation. 

We found, on the contrary to studies carried out by Shin et al. (2005) who found that individuals with bipolar disorder experience a higher seasonality variation, no differences among diagnosis in seasonality scores [44]. This could be due to the fact a large portion of our sample has a history of maintenance therapy; unipolar subjects with a high rate of depressive recurrences are treated with maintenance therapy (mainly with SSRI), while bipolars are for the most part on long-term lithium treatment, which, as mentioned previously, may alter and reduce seasonal fluctuations. 

Limitations of our study include that the use of retrospective questionnaires (such as the SPAQ+) always raise the possibility of recall bias. Furthermore, we have a lack of information about the lighting conditions to which subjects were exposed in their usual environment and the study period was relatively short in length and multiple years of data may be required to detect statistically significant seasonal effects. Another major limitation of our study is that we did not collect a control sample. Finally, as we have seen, sleep, light, and seasonality seem to be three interconnected features that lie at the basis of chronobiology that, when altered, have an important effect both on the psychopathology and on the treatment depression. These findings, paired with future acquisitions, will be useful in the clinical practice, both for prevention and for the manipulation of these features for a therapeutic aim. 

## Figures and Tables

**Table 1:** Demographic and clinical characteristics of the sample.

Tot. *n* = 113	Unipolar	Bipolar
	*N* = 46	*N* = 67
	mean ± SD	mean ± SD
Age	54.98 ± 11.88	51.32 ± 13.20
Education years	11.83 ± 4.24	12.05 ± 4.31
Age of onset	34.00 ± 14.44	31.72 ± 13.47
Duration of Illness (years)	20.98 ± 12.88	18.98 ± 12.15
Total no. of episodes	5.39 ± 4.86	8.36 ± 7.91
Depressive episodes	5.21 ± 4.35	5.20 ± 5.32
Manic episodes	/	4.63 ± 12.45
Duration of euthymia (months)	29.93 ± 37.19	30.57 ± 35.60
Duration of maintenance Therapy (months)	40.36 ± 50.91	54.51 ± 48.84

**Table 2:** *t*-test analysis between unipolar and bipolar patients.

	Unipolars (*N* = 46)	Bipolars (*N* = 67)	*t* value	*P*
	Mean ± SD	Mean ± SD		
Total Number of episodes	5.39 ± 4.86	8.36 ± 7.91	−2.27	0.025
Duration of maintenance therapy (months)	40.36 ± 50.91	54.51 ± 48.83	−1.25	0.214
Episodes during maintenance therapy	0.26 ± 0.77	0.68 ± 1.50	−1.67	0.097
GSS	8.39 ± 4.88	8.22 ± 4.65	0.18	0.854
Sleep disturbance	37.39 ± 25.04	24.32 ± 20.40	3.01	0.003
Snoring	38.67 ± 29.05	48.91 ± 39.96	−1.47	0.145
Short of breath	19.57 ± 24.03	14.38 ± 23.49	1.13	0.260
Sleep adequacy	36.00 ± 26.26	27.34 ± 27.62	1.65	0.101
Sleep somnolence	31.39 ± 20.54	38.59 ± 27.80	−1.49	0.139
Sleep hours	7.26 ± 1.73	7.41 ± 1.36	−0.52	0.604
Recurrence index	2.87 ± 2.95	4.36 ± 2.99	−0.55	0.010

**Table 3:** *t*-test analysis between unipolar and bipolar patients that have a family have a family history for mood disorders versus those that do not.

	No family history (*N* = 34)	Family history (*N* = 78)	*t* value	* P*
	Mean ± SD	Mean ± SD		
Age	53.18 ± 14.39	52.46 ± 12.03	0.27	0.786
Education	11.21 ± 4.40	12.37 ± 4.14	−1.34	0.181
Age of onset	39.79 ± 14.82	30.18 ± 11.36	3.74	0.000
Duration of illness (years)	14.15 ± 8.26	22.14 ± 13.23	−3.25	0.002
Number of depressive episodes	3.35 ± 2.21	6.00 ± 5.48	−0.34	0.009
Number of manic episodes	1.29 ± 1.49	3.33 ± 11.75	−2.66	0.319
Total number of episodes	4.59 ± 3.13	8.19 ± 7.83	−1.00	0.011
Duration of maintenance therapy (months)	53.73 ± 52.33	48.23 ± 49.06	−2.58	0.636
GSS	6.85 ± 4.16	8.90 ± 4.87	−2.13	0.035

**Table 4:** *t*-test analysis between unipolar and bipolar patients that are in maintenance therapy with lithium salts versus patients that are not.

	No lithium	Lithium	*t* value	*P*
	(*N* = 38 unipolars, 17 bipolars) (*N* = 55)	(*N* = 5 unipolars, 50 bipolars) (*N* = 55)		
	Mean ± SD	Mean ± SD		
Sleep disturbance	36.28 ± 24.11	23.30 ± 20.64	3.03	0.003
Short of breath	24.72 ± 28.54	8.361 ± 13.71	3.83	0.000
Sleep adequacy	39.93 ± 27.81	22.00 ± 23.76	3.63	0.000
Sleep somnolence	39.83 ± 23.83	31.33 ± 25.98	1.79	0.060

**Table 5:** *t*-test analysis between morningness and eveningness patients.

	Eveningness types (*N* = 16)	Morningness types (*N* = 67)	*t* value	* P*
	Mean ± SD	Mean ± SD		
Sleep disturbance	40.17 ± 28.07	25.61 ± 18.71	2.52	0.010
Short of breath	18.75 ± 17.08	17.42 ± 27.22	0.19	0.853
Sleep adequacy	47.50 ± 33.37	28.09 ± 26.24	2.53	0.013
Sleep somnolence	48.58 ± 28.873	35.33 ± 25.46	1.83	0.071
